# Targeting *Aspergillus* allergen oryzin with a chemical probe at atomic precision

**DOI:** 10.1038/s41598-023-45028-z

**Published:** 2023-10-20

**Authors:** Olivia N. Pattelli, Dinh Dinh Ly Diec, Wanting Guo, Silvia Russi, Daniel Fernandez

**Affiliations:** 1 Sarafan ChEM-H, Stanford University, Stanford, CA 94305 USA; 2 Macromolecular Structure Knowledge Center, Stanford University, Stanford, CA 93405 USA; 3 Structural Molecular Biology Group, Stanford Synchrotron Radiation Lightsource (SSRL), SLAC National Accelerator Laboratory, Menlo Park, CA 94205 USA

**Keywords:** Biochemistry, Biological techniques, Biophysics, Chemical biology, Computational biology and bioinformatics, Molecular biology, Structural biology, Diseases, Molecular medicine, Chemistry, Physics

## Abstract

We report the molecular basis of *Aspergillus fumigatus* oryzin, allergen Asp f 13, or alkaline proteinase ALP1, containing the sequence motif His–Asp–Ser of the subtilisin family, structure, and function at atomic detail. Given the resolution of the data (1.06 Å), we use fragment molecular replacement with ideal polyalanine α-helices to determine the first crystal structure of oryzin. We probe the catalytic serine through formation of an irreversible bond to a small molecule compound, specifically labeling it, describing the amino acid residues performing the catalytic function. Defined by a self-processed pro-peptide, the active site architecture shapes up pocket-like subsites that bind to and unveil the S1′–S4′ substrate binding preferences. We use molecular modeling to dock a model of the pro-peptide in the S1–S4 region and to dock collagen along the active site cleft. Opposite to the face harboring the catalytic serine, the enzyme binds to a calcium ion in a binding site created by backbone flipping. We use thermal unfolding to show that this metal ion provides structural stability. With no known host inhibitor identified thus far, this structure may hasten the progress of developing new therapeutic agents for diseases caused by pathogenic fungi.

## Introduction

*Aspergillus* spp. are microorganisms commonly found in the environment that produce airborne fungal spores. Several hundred spores can be inhaled and eliminated per day without harm to the host; however, in individuals predisposed to infection through lung disease and in patients undergoing cancer chemotherapy or immunosuppressive therapy following organ transplants, aspergilli can cause invasive pulmonary aspergillosis, which can be a life-threatening condition^1,2^. Several studies have shown that patients with severe COVID-19 had a high risk of developing invasive fungal infections, including aspergillosis^3^. Among aspergilli, the World Health Organization has designated *Aspergillus fumigatus* as a critical priority pathogen^4^. Azoles are the first choice of drug for the treatment of aspergillosis; however, reports of antifungal resistance are increasing in the literature^5,6^ such that up to 20% of *Aspergillus* isolates exhibit de novo resistance to commonly used antifungal agents^7^. Aspergillosis is an increasingly difficult condition to diagnose and treat^8,9^. Recent data suggests that the pathogen's genetic pool for key metabolic and catabolic enzymes in human infection is more versatile than originally thought^10^. More research into available molecular targets will help us understand the complexities of this organism and its relation to host virulence, drug resistance, and target characterization with the goal of developing new therapeutics.

Fungal colonization of the lungs requires a vast supply of hydrolytic enzymes. *Aspergillus* spp. degradome includes 111 peptidase genes (about 1% of the genome) coding for 14 different types of peptidases^11^. The most studied of such peptidases is the alkaline serine peptidase oryzin (also known in the medical literature as allergen Asp f 13, alkaline proteinase Alp1, and elastinolytic serine proteinase). By a conserved sequence motif, and the Asp–His–Ser triad arrangement, oryzin belongs to the SB clan, S8A subfamily of subtilisins^12^. Initially, oryzin clinical isolates were found to hydrolyze elastin, a key protein component of the connective tissue, and fibrinogen, a blood-circulating glycoprotein, hence mechanistically this enzyme was characterized as elastinolytic or fibrinogenolytic^13,14^. Different *Aspergillus* strains including *A. fumigatus*, *A. flavus*, *A. nidulans*, and *A. terreus* showed a strong digestion of collagen mediated by abundantly secreted oryzin when grown in a collagen medium^15,16^. Another study showed oryzin efficiently cleaved complement components C3, C4, C5, and C1q, as well as immunoglobulin G, and that this was the only serine protease in culture supernatant performing this activity, such that it could be prevented by abrogating the oryzin gene^17^. Additionally, oryzin isolated from fungal spores was shown to detach cultured Vero cells suggesting oryzin's ability to remove attachment without impairing the cells^18^. In patients with asthma, *Aspergillus* may cause a spectrum of conditions from allergic bronchopulmonary aspergillosis to severe asthma with fungal sensitization. In murine models of fungal asthma, oryzin was found as an essential mediator for the recruitment of inflammatory cells and remodeling of the airways^19^, disrupting airway smooth muscle cell-extracellular matrix interactions, and eroding epithelial cell junctions^20–22^. Using a deletion mutant of the oryzin gene, Namvar et al.^19^ found that oryzin was indispensable for the initiation and progression of fungal asthma. In summary, oryzin activity on host proteins during infection serves as a promising route for the development of diagnostic tools and inhibitors to target this peptidase. However, the molecular basis for substrate recognition is largely unknown and is difficult to rationalize based on primary sequence alone. To provide a deeper insight into the function and structure of the *Aspergillus* secreted serine peptidase oryzin, we determined its first three-dimensional structure by X-ray crystallography. We obtained complexes with its pro-peptide segment, defined the substrate binding pockets, a tetrahedral irreversible adduct with the catalytic serine, captured the interactions between the key residues and calcium in catalysis, defined the metal ion binding site that provides structural stability.

## Results

### Oryzin folds as an α/β-hydrolase enzyme used by prokaryotic and eukaryotic cells

**Figure 1:**
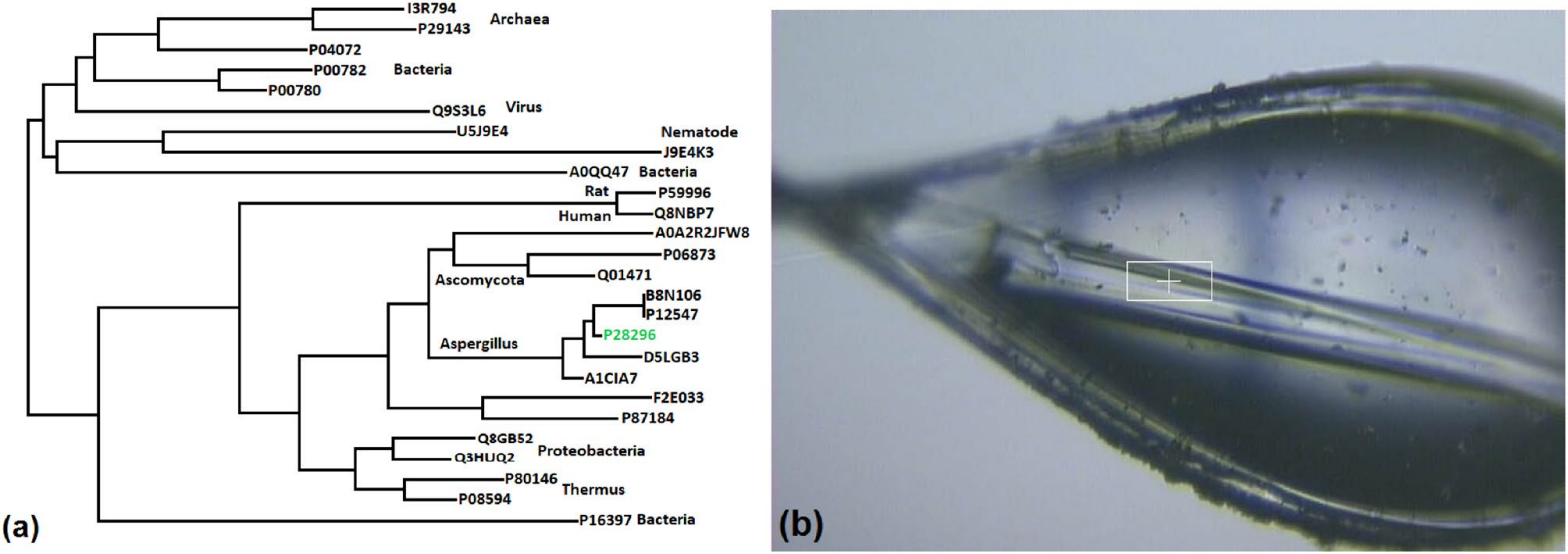
Distribution of subtilisins among taxonomic kingdoms. (**a**), phylogenetic distribution. Codes are from UniProtKB^27^. P28296, *Aspergillus fumigatus* oryzin (this work, in green). (**b**), oryzin crystallized as a long rod (dimensions 10 × 20 × 400 μm) that was used for a helical data collection strategy with microfocused X-ray beam along the longest dimension of the crystal. Box size dimensions: 50 × 15 μm.

Proteolysis is the mechanism by which peptide bonds in proteins are broken, creating new peptides and aminoacids. This splitting reaction is facilitated by peptide-degrading enzymes called proteases or peptidases or peptide hydrolases. Peptidases differ by the nature of the active group that drives the hydrolytic reaction, with serine peptidases as the most abundant in nature, followed by metallo, cysteine, aspartate and threonine proteases, respectively^23^. Typically, serine peptidases are synthesized as large multi-segmented polypeptides (zymogens) that are activated auto-catalytically and/or exogenously. Previous work with clinical isolates of oryzin-producing spores identified the Asp–His–Ser catalytic signature typical for serine peptidases of the subtilisin type^24–26^. Given the importance of serine proteases in key biological reactions carried out both inside and outside the cell, they are present among all taxonomic kingdoms, from archaea to animals (Fig. 1a). Specifically, the extracellular activity of oryzin is being investigated for its involvement in aspergillosis and fungal-associated diseases. The reactive sites and mode of substrate binding of oryzin are largely unknown and are difficult to predict based on sequence alone. To structurally characterize oryzin, we recombinantly produced it in bacterial cells and purified it to homogeneity. We then obtained crystals of oryzin (Fig. 1b), performed X-ray diffraction, and determined the three-dimensional structure.

**Figure 2:**
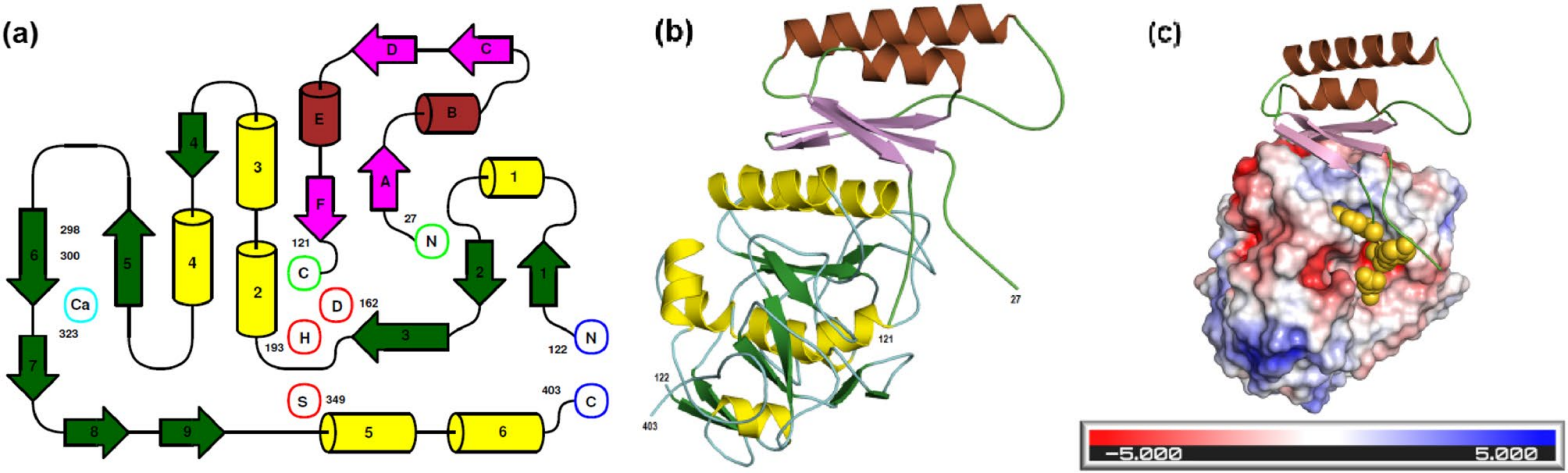
Oryzin is a binary complex resulting from proteolysis and trapping of cleavage product. (**a**), topological diagram of oryzin indicating secondary structure elements and key residues. Catalytic domain (green and yellow), pro-domain (magenta and brown). N– and C-termini, catalytic triad, and divalent metal binding site are marked as circles (blue, green, red, sky-blue). Figure prepared with Topdraw^29^. (**b**), cartoon depicting oryzin with secondary structure elements colored as in panel (**a**). (**c**), electrostatic potential surface of oryzin. Pro-domain shown as cartoon, with residues 118–121 depicted as space-filling model. Orientation shifted 45 degrees to facilitate view to the catalytic site (center of figure). Figures in panels (**b**) and (**c**) prepared with Pymol^30^.

The crystal structure of oryzin consists of two polypeptide chains, the pro-peptide (residues 27–121) and the catalytic domain (residues 122–403), originating from a single polypeptide precursor (Fig. 2a). Cleavage at the junction between Asp121–Ala122 releases a new N-terminal segment that displaces to a new position in the mature structure. Upon bond breaking the initially contiguous amino acids are separated by 36 Å. The segment containing residues 122–132 pivots on Gly132 to its final position away from the catalytic site and rebuilds to form the new β-strand 1. This process is similar to the maturation of *Aspergillus fumigatus* secreted zinc-metalloprotease^28^, where the P1′-peptide refolds to a new secondary structure element in a new location. The oryzin pro-peptide fold consists of two α-helices packed against a β-sheet with a C-terminal coil partially occupying the catalytic cleft (Fig. 2b). The oryzin pro-peptide is attached to the catalytic domain by non-covalent interactions mediated by C-terminal residues, Trp118-Leu119-Tyr120-Asp121 (this will be discussed in a later section). Oryzin displays one face crossed by a long cleft that is predominantly negatively charged at the entry of the catalytic site (Fig. 2c). As shown below, these charges and surface properties will help position oryzin to bind to its substrate.

**Figure 3:**
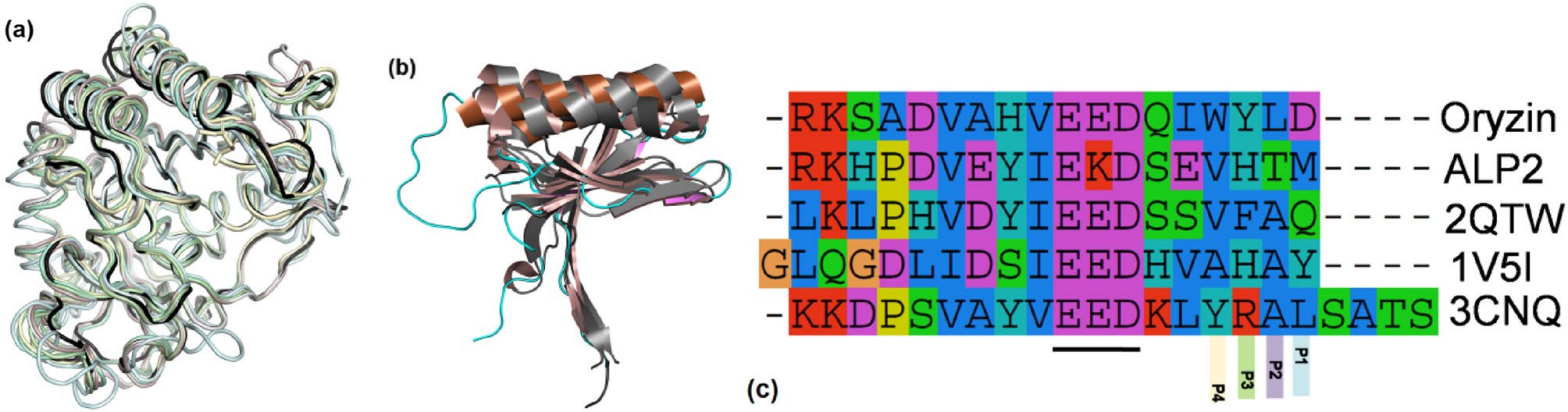
Structure of oryzin catalytic domain and pro-peptide show fold similarity to enzymes from different origins. (**a**), ribbon representation of the catalytic domain of oryzin (black) and subtilisin-like enzymes (tones of green; shown PDB IDs: 5z6o, 3f7o, 6kkf, 2b6n, 4dzt and 2qtw (except that the C-terminal 250 residues in human kexin were omitted for clarity). Same orientation as in Fig. 2, c. Cα atoms aligned: 5z6o (276 residues, rmsd 0.96 Å); 3f7o (273 residues, rmsd1.01 Å); 6kkf (266 residues, rmsd 1.06 Å); 2b6n (264 residues, rmsd 0.85 Å); 4dzt (268 residues, rmsd 0.97 Å); and 2qtw (251 residues, rmsd 1.41 Å). (**b**), cartoon representation of the pro-peptide (colored brown, magenta and cyan) overlay to equivalent regions (grey) in human proprotein convertase subtilisin/kexin type 9 [PDB ID: 2qtw; (80 residues, rmsd 1.84 Å)] and the bacterial subtilisin from *Bacillus amyloliquefaciens* [PDB ID: 3cnq; (62 residues, rmsd1.71 Å)] and a natural subtilisin inhibitor from the fungus *Pleurotus ostreatus* [PDB ID: 1v5i; (72 residues, rmsd 2.07 Å)]. (**c**), detail of alignment of oryzin C-terminal tail to subtilisin-like pro-domains and a peptidase inhibitor (from top to bottom: oryzin, ALP2 (Uniprot P81784, *A. fumigatus* intracellular alkaline serine protease), 2qtw, 1v5i, and 3cnq). Underlined, conserved negatively charged residues. P1–P4 residues N-terminal to the scissile bond. Alignment made with Seeview^38^.

Oryzin has a globular fold that conforms to the α/β three-layered sandwich type resembling eukaryotic and prokaryotic subtilisin-like peptidase enzymes (Fig. 3a). A 3D structural comparison using the DALI server^31^ top-score hits were with a subtilisin protease from *Penicillium cyclopium*, a plant-infecting fungus [PDB ID: 5z6o;^32^]; a cuticle-degrading protease from *Paecilomyces lilacinum*, an opportunistic fungus commonly found in the soil [PDB ID: 3f7o;^33^]; and, a proteinase K-like enzyme from *Parengyodontium album* (also known as *Tritirachium album*, *Beauveria alba*, and *Engyodontium album*), a causative agent of mycoses [PDB ID: 6kkf;^34^]. Further, bacterial enzymes such as a proteinase K-like from *Serratia* spp. [PDB ID: 2b6n;^35^] and the aqualysin from *Thermus aquaticus* [PDB ID: 4dzt;^36^] are highly similar. In addition, the structure aligns to the human pro-protein convertase/subtilisin kexin type 9 [PDB ID: 2qtw;^37^] at a rmsd 1.4 Å over 251 Cα atoms.

There is also significant structural homology in the pro-domain regions of human and bacterial subtilisin despite low sequence similarity (< 20%) [Fig. 3b]. These include the pro-domain of the proprotein convertase subtilisin/kexin type 9 (PDB ID: 2qtw) and a bacterial subtilisin from *Bacillus amyloliquefaciens* [PDB ID: 3cnq;^39^]. Interestingly, it also is similar to a natural subtilisin inhibitor from the fungus *Pleurotus ostreatus* [PDB ID: 1v5i;^40^]. Contained in the C-terminal tail of oryzin, there is a common motif involving a stretch of negatively charged residues around position 113 (Fig. 3c). Within this acidic region, His111 stacks to Tyr262, while the carboxyl of Glu113 hydrogen bonds to the main chain N of Tyr262 and the Oγ of Ser261. Similarly, Gln116 is hydrogen bonded to the main chain carbonyl atoms of Gly259 and Tyr260. In summary, these interactions anchor and orient the C-terminal tail toward the catalytic site. Next, to precisely delineate the catalytic active site cleft architecture, we use this crystal structure in combination with molecular modeling to determine the structural basis for substrate recognition.

### Oryzin substrate binding specificity

**Figure 4:**
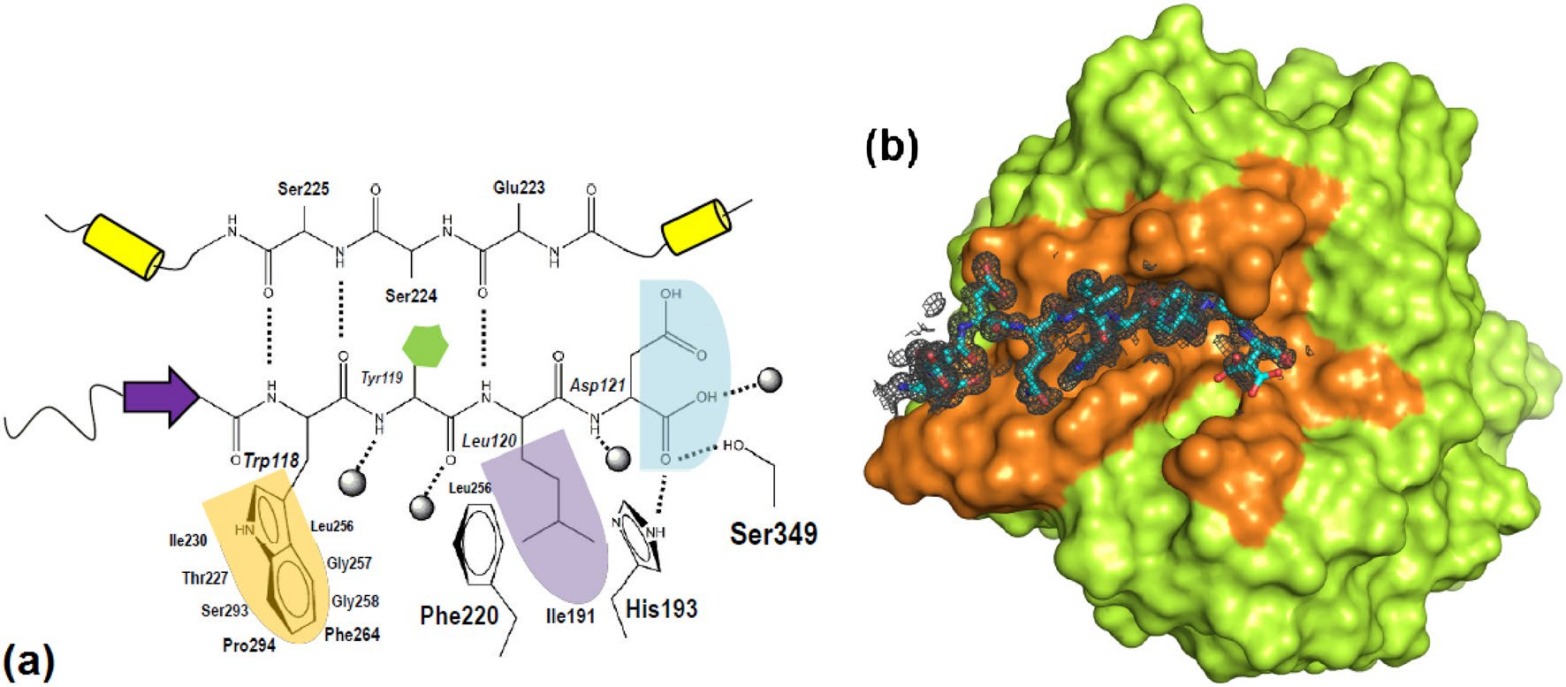
Orizin pro-peptide C-terminal tail binding pocket. (**a**), schematic diagram showing pockets S4 to S1 from left to right (colored yellow, green, violet and sky blue). (**b**), pro-peptide segment 113–121 (sticks) harboring P4–P1 residues Ile117-Trp118-Tyr119-Leu120-Asp121). At center of figure, Asp121 is in two split orientations. Oryzin shown as a surface (lemon), with pro-peptide-interacting residues (under 4 Å distance) in orange. Electron density map contoured at the 1.0-σ level (mesh in gray).

About 12% of the accessible surface area of oryzin catalytic domain (~ 1220 Å^2^ out of total ~ 10,080Å^2^) is occupied by pro-domain atoms. Although both the N– and C–termini are unstructured segments, the C-terminal residues Trp118-Leu119-Tyr120-Asp121 from the pro-peptide remain bound in the catalytic cleft. They reveal the S1 to S4 substrate binding pockets in oryzin (Fig. 4a).

Trp118 is buried deep in a hydrophobic pocket lined by several small amino acid side chains where strong hydrogen bonds are established between the main chain atoms of this residue and Ser225 (Fig. 4b). The bulky aromatic moiety of Trp118 forms hydrophobic interactions with Leu256, Ser293, Pro294, Phe264, Ile230, Thr227, Gly257 and Gly258. The Tyr119 side chain makes a hydrophobic contact to Ser224 but mostly faces outward towards the solvent and further side chains in the pro-peptide. Here, the bulky side chain of Phe220 protrudes, further constraining contacts in S3, while most of interactions are through water molecules to the main chain N and O of Gly257. Leu120 is sandwiched between Phe220 and His193 with extra hydrophobic interactions to Leu256 and Ile191. In addition, the N of the amide bond of Leu120 hydrogen bonds to the main chain O of Glu223. Finally, the side chain of P1, Asp121, faces outwards towards the solvent while its main chain O (now the new COOH terminus) is hydrogen bonded to both Ser349 and His193. A further hydrogen bond is mediated through a water molecule to the main chain O of Ser346. An overlay with the human proprotein convertase subtilisin/kexin type 9 (PDB 2qtw; sequence ID 19%, 80 aligned Cαs) shows that the equivalent side chains occupying the S4–S1 subsites in the human pro-peptide are valine, phenylalanine, alanine, and glutamine. The bacterial serine proteinase inhibitor from the mushroom *P. ostreatus* (PDB 1v5i; sequence ID 20%, 70 aligned Cαs) shows alanine, histidine, alanine, and tyrosine. In the model serine proteinase from *B. amyloliquefaciens* (PDB 3cnq; sequence ID 28%, 67 aligned Cαs), the P4–P1 residues are tyrosine, arginine, alanine, and leucine. The comparison to structurally characterized counterparts indicate that substrate binding preferences are for subsites: (S4), bulky aromatic/aliphatic; (S3), large side chains, aromatic or otherwise; (S2), small aliphatic side chain; and (S1), large side chains, aliphatic or not. Interestingly, subsites S4 and S2 show a preference to bury aromatic/aliphatic side chains while also promoting strong hydrogen bonding interactions; in contrast, subsites S3 and S1 seem open to accommodate side chains with lower selectivity. In summary, these binding interactions illustrate a distinct pattern of substrate-binding preferences among different subtilisins. Next, we use molecular modeling to explore the C-terminal subsites and the binding of an oryzin protein substrate.

### Molecular modeling—a structural rationale for substrate recognition

To delineate oryzin’s binding pockets beyond the S1 site, we performed molecular modeling using a peptide fragment with adjacent P1′–P4′ residues extending the pro-domain from C-terminal residue Asp121.

**Figure 5:**
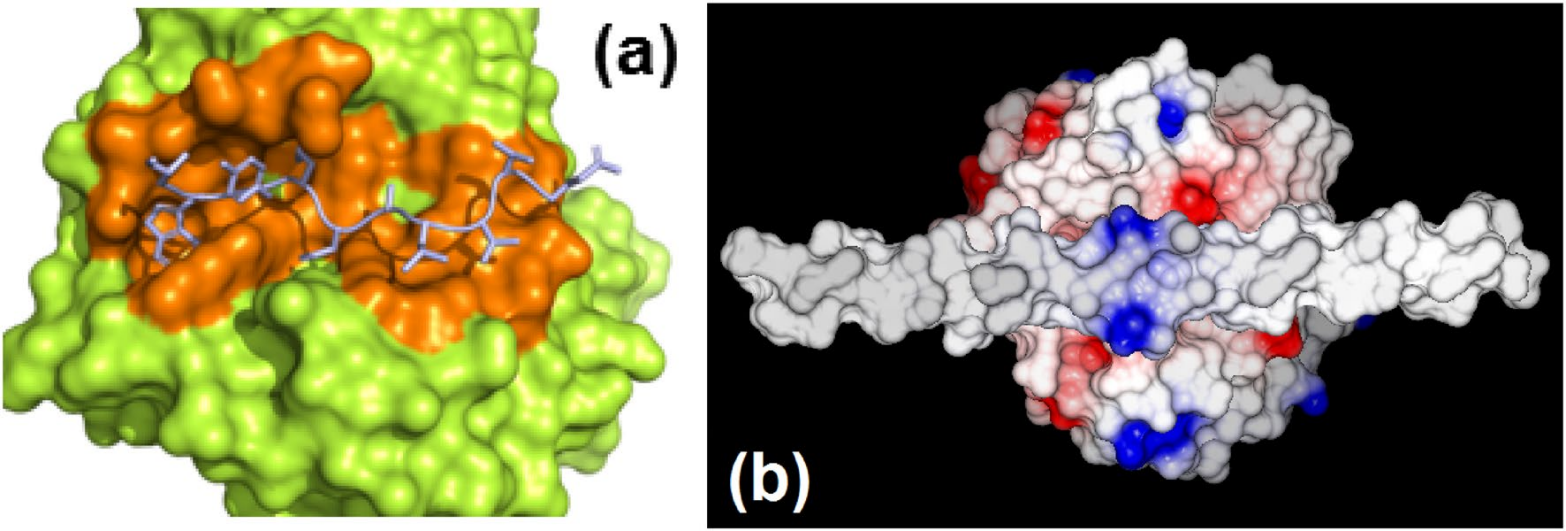
The catalytic cleft extends 32 Å or about a 9-aminoacids-long stretch. (**a**)*,* model of a short peptide encompassing residues 118–126 (sticks representation in gray) binding to oryzin (surface in lime, residues interacting with the peptide highlighted orange). (**b**)*,* model of collagen docked to oryzin. Oryzin electrostatic potential surface as in Fig. 2.

Our model shows a transition from pocket-like on the non-prime subsites to a shallow plain on the prime subsites (Fig. 5a). Covering one face of the enzyme, the catalytic cleft is an elongated groove spanning ~ 32 Å from S4 to S4′ (~ 9 peptide bonds). The P1–P1′ scissile bond (Asp121–Ala122) is 3.8 Å from the Oγ oxygen of the catalytic serine (Ser349). The P1′ and P2′ side chains, Ala122 and Leu123, make hydrophobic contacts with the side chains of Ile345 and Trp335 and form hydrogen bonds to main chain atoms anchoring them. These small side chains would be small enough to fit the scissile bond closer to the catalytic serine. Similarly, two further small residues in the sequence, Thr124 (P3′) and Thr125 (P4′), would be buried in a depression at Ser346 and Gly347, promoting hydrogen bonding interactions between main-chain atoms. The prime residues are exposed to the solvent more than the non-prime ones, explaining why the new C-terminal segment at residue 122 springs out of the catalytic site after cleavage while the N-terminal residue 121 remains bound. To study the binding between oryzin and its substrate collagen, we used molecular modeling to dock collagen to an oryzin structure that does not contain the pro-domain. Collagen is a neutrally charged polypeptide, except for a segment of positively charged arginines (Fig. 5b, center of figure in blue) that would interact with the negatively charged catalytic pocket in oryzin. In summary, the architecture of oryzin catalytic cleft facilitates binding of extended protein substrates along the cleft's axis, exposing the scissile peptide bond to the catalytic machinery. We next use a chemical probe to confirm the identity of the catalytic active site residue and the oxyanion binding site in oryzin.

### *The hydrogen bonding in the Asp*–*His*–*Ser catalytic triad and the oxyanion binding site*

**Figure 6:**
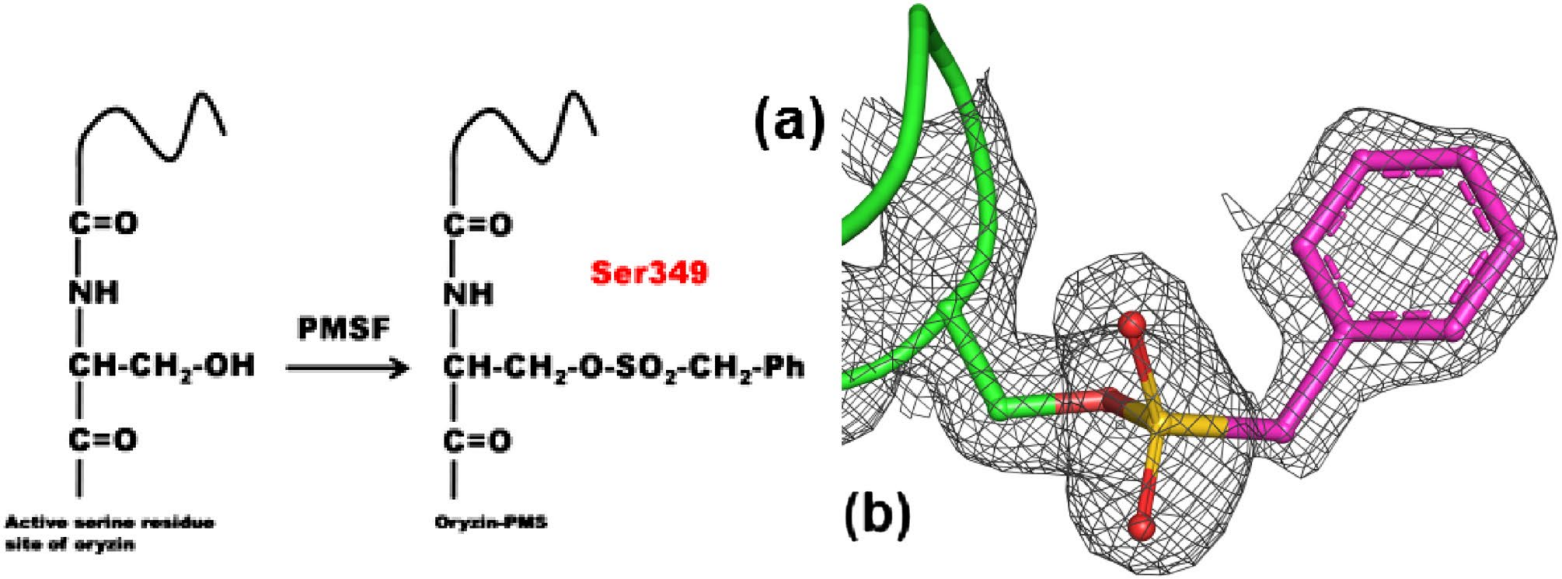
Oryzin-PMS covalent bonding crystal structure probes Ser349. (**a**), chemical scheme for reacting a serine to the irreversible inhibitor PMSF. (**b**), Ser349 in oryzin-PMS crystal structure. The covalent bonding to Ser349 is verified. Atoms rendered as sticks in green (carbon, protein), magenta (carbon, PMS), gold (sulfur), and red (oxygen). Omit-calculated electron density map (black mesh) contoured at the 1.0-σ level.

The mechanism of the catalytic triad in serine peptidases is highly studied and well understood. In serine peptidases, the primary hydroxyl group of the active site serine residue acts as the nucleophile. The histidine activates serine by acting as a base and accepting the serine's single proton. The polarized hydroxyl group attacks the carbonyl carbon resulting in a negatively charged tetrahedral intermediate that is stabilized by a hydrogen bond from a nearby asparagine that constitutes the oxyanion binding site. Finally, the amine group leaves and the tetrahedral intermediate is broken by a histidine-activated water molecule^41^. We use phenylmethanesulfonyl fluoride (PMSF), a chemical compound that sulfonylates serine to form a stable adduct, to introduce an oryzin-PMS crystal structure (Fig. 6a). The covalent attachment between the inhibitor and the Oγ of Ser349 shows that the enzyme is fully active and confirms the identity of Ser349 as the oryzin catalytic reactive serine (Fig. 6b).

**Figure 7:**
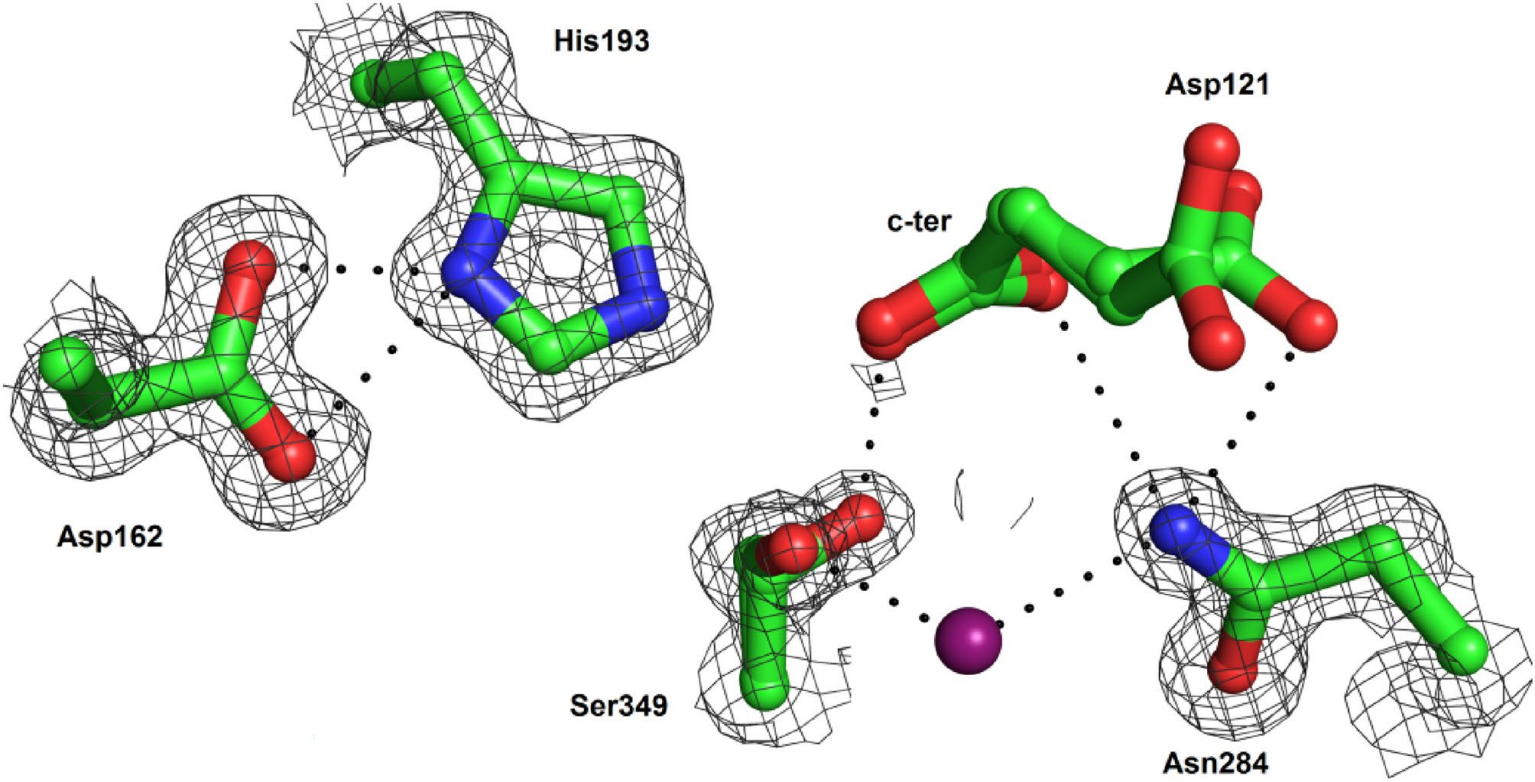
Hydrogen bonding interactions in oryzin catalytic site. Atoms rendered as sticks in green (carbon), blue (nitrogen), and magenta (oxygen). Hydrogen bonds shown as dotted lines (black). Electron density map (gray mesh) for catalytic site residues contoured at the 1.0-σ level.

Next, we analyze the oryzin Asp–His–Ser triad hydrogen bonding interactions (Fig. 7). The pair His193 and Asp162 are at hydrogen bonding distance sharing the Nδ1 hydrogen. We hypothesize that histidine may be in its ionized form. For this to occur, the Nε2 atom of His193 would have to abstract a hydrogen atom from Ser349. These atoms are 3.3 Å apart, at hydrogen bonding distance, but neither of the two split orientations of the hydroxyl group of Ser349 (this side chain is disordered and modeled in two sites) points in the direction of the histidine. One of the two disordered oxygens of Ser349 is hydrogen bonded to the C-terminal carboxyl of Asp121 (residue disordered and modeled at two alternative sites). The second disordered oxygen of Ser349 is hydrogen bonded through a water molecule to the nearby Asn284. Asn284 hydrogen bonds to the C-terminal carboxyl and the side chain of Asp121. Asn284, a conserved residue in the subtilisin family, is the oxyanion binding site in oryzin.

The side chain hydroxyl group of Ser349 builds a hydrogen binding network with the C-terminal carboxyl group of Asp121 and the side chain amide nitrogen of Asn284 (through a water molecule). In summary, the geometry around base His193, hydrogen bonded to Asp162 farther from the catalytic Ser349 Oγ, and the latter hydroxyl group pointing away His193 in the direction of Asn284 and the new C-terminal carboxyl, would suggest that the enzyme's structure represents an end-product state. We then use a peptide substrate to analyze the enzyme's activity after its activation through chemical unfolding.

### Enzyme activity

**Figure 8:**
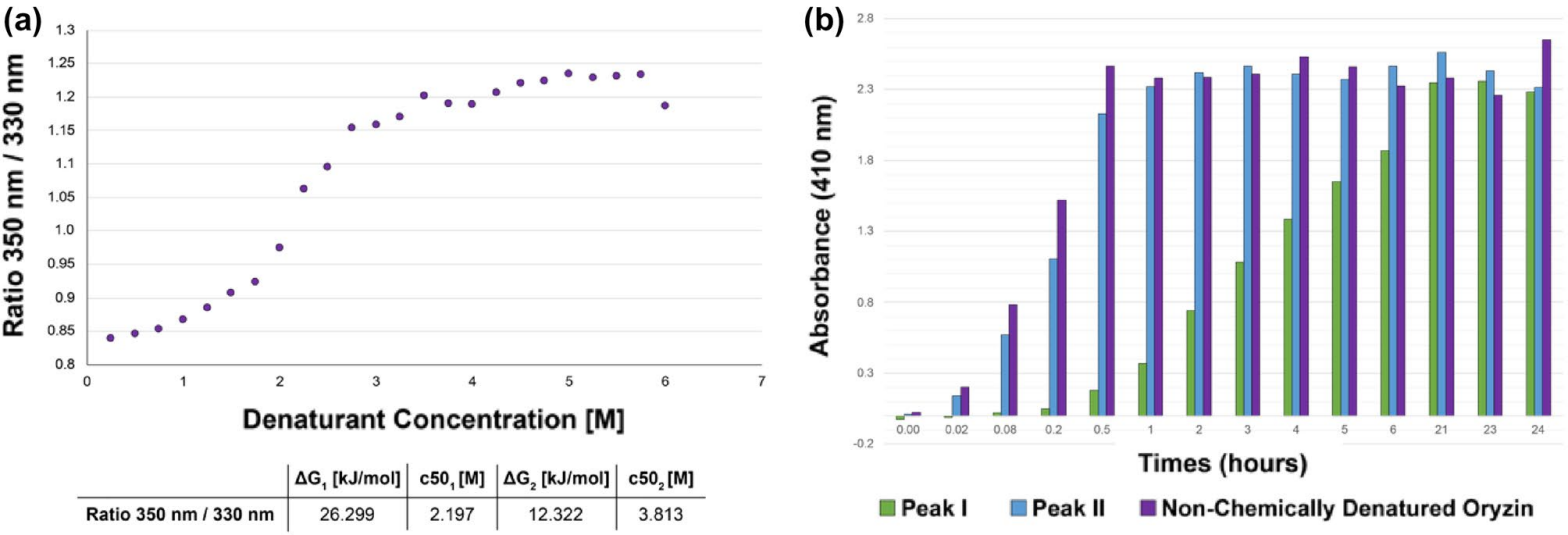
Chemical unfolding of oryzin leads to cleavage of the pro-peptide and slowed enzymatic activity. (**a**) Chemical unfolding of oryzin using increasing concentrations of GuHCl as shown as a fluorescence ratio 350 nm/330 nm. (**b**). Enzyme–substrate activity assay using peak I, peak II, and non-chemically denatured oryzin samples. The substrate used was Suc-Ala-Ala-Pro-Phe-pNA. Activity of each sample was measured at 410 nm.

Oryzin is expressed as a single polypeptide that can self-cleave the pro-peptide portion of the protein. The cleaved pro-peptide remains bound, partially occupying the catalytic cleft and hindering access to the catalytic Ser349. Previous work established that chemical denaturants, such as urea, can be used to dissociate an enzyme's catalytic domain from its pro-peptide^42^. Though urea is a commonly used chemical denaturant, solutions of urea are not stable and are subject to decomposition. To circumvent this, we use guanidine hydrochloride (GuHCl). To determine the ideal denaturant concentrations for purification, we perform chemical unfolding of oryzin with differential scanning fluorimetry (DSF) monitoring changes in fluorescence as a function of GuHCl concentration. We determined that oryzin has two major unfolding events at 2.2 M and 3.8 M GuHCl (Fig. 8a). Due to the shorter length of the pro-peptide sequence and the nature of its tertiary structure (about 50% of its fold is loops or unstructured regions; Fig. 2b), we hypothesized that this portion of the zymogen will unfold first, suggesting that denaturing at this concentration of GuHCl is sufficient for the unfolding and dissociation of the pro-peptide and the catalytic domain of oryzin. Oryzin was expressed, the lysate was incubated with 2.2 M GuHCl, and then purified using immobilized metal affinity chromatography (IMAC) and size exclusion chromatography (SEC) to isolate two distinct species as shown by SDS-PAGE chromatography (Supplemental Figure S2). Peak I contained a major band with an estimated molecular weight of 28 KDa, and a 21 kDa band suggesting one or more events of hydrolytic cleavage. Peak II contained a major band with an apparent molecular weight of 33 kDa, indicating a distinctly cleaved oryzin containing the two minor bands of 28 and 21 kDa. This is in agreement with several previous works that isolated oryzin from hospital samples predominantly as 32–33 KDa products and degradation products at 28 and 23 KDa and smaller^13–15,26^. We then use a previously identified synthetic substrate such as Suc-Ala-Ala-Pro-Phe-pNA (Suc-AAPF-pNA)^13^ for the determination of oryzin enzymatic activity of peaks I and II and a non-chemically-denatured sample (33 KDa, see Supplemental Figure S1).

We found that while all three samples reach similar hydrolytic activity after 24 h (Fig. 8b), the peak I sample does so more slowly than the Peak II and the non-denatured sample (green vs. blue/purple bars). This suggests that chemical denaturation of the protein results in the dissociation of the pro-peptide with concurrent enzyme cleaving itself to lower molecular weight products, weakening its enzymatic activity. An alternative could be that degradation products could remain bound and block enzymatic activity.

### Calcium-binding stabilizes the enzyme through backbone remodeling

**Figure 9:**
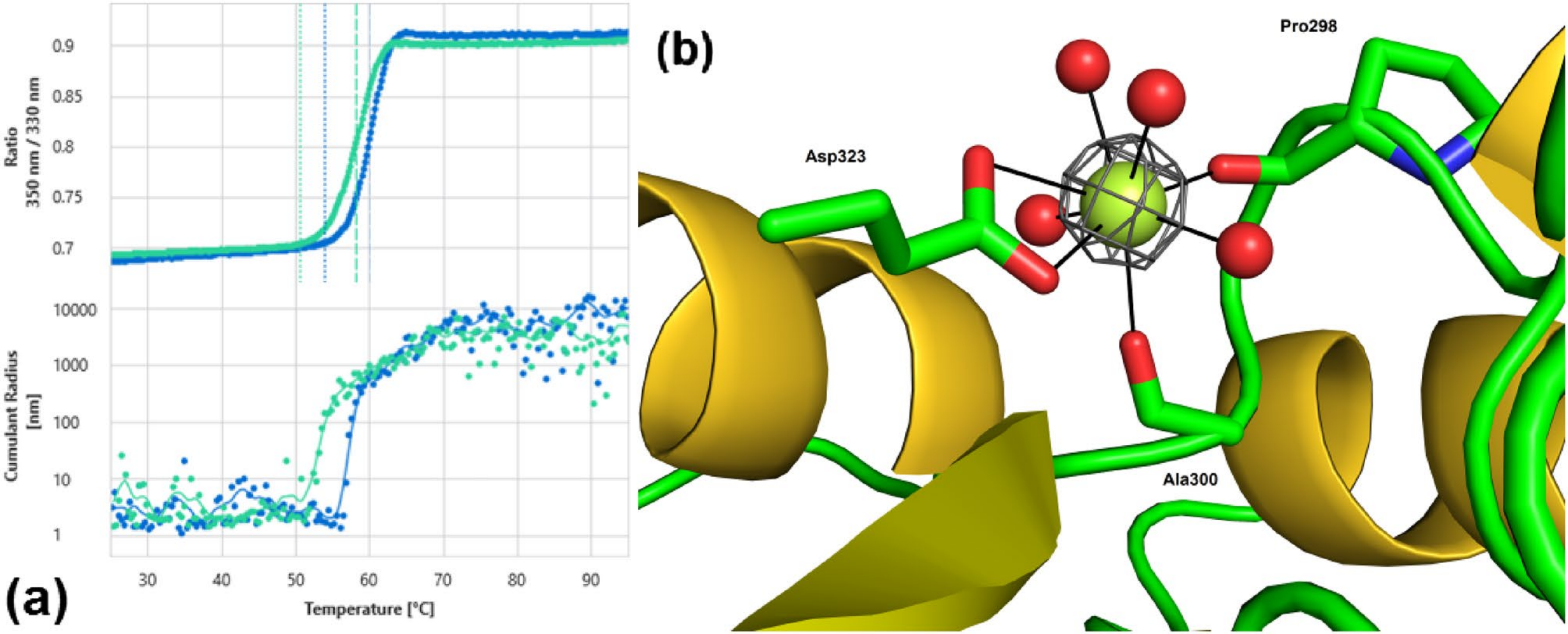
Oryzin thermal unfolding is slowed by calcium binding to a pocket created by backbone flipping. (**a**), nanoDSF trace of oryzin melting. On increasing temperature, in the absence of calcium (green) oryzin melts (*top panel*) and aggregates (*bottom*) at a lower temperature than in the presence of calcium (blue). (**b**), calcium binding site in oryzin. Shown as sticks Pro298, Ala300, and Asp323 (carbon atoms, green; nitrogen, blue; oxygen, red). Calcium is as a sphere (lemon) with bonding interactions (solid lines in black). Anomalous electron density map (grey mesh) contoured at the 4.0-σ level.

Earlier work demonstrated that ethylenediaminetetraacetic acid (EDTA), a compound typically used for sequestering metal ions, could render oryzin inactive^14^. However, other researchers found EDTA did not inhibit activity, but divalent metal ions, like calcium, had an activity-modifying effect^15^. To better understand the role of divalent metals on oryzin, we use differential scanning fluorimetry (DSF) to measure the unfolding of oryzin in the presence of calcium, by monitoring changes in fluorescence as a function of temperature. We found that oryzin unfolds at a lower T_m_ (the T_m_s are 58.38 (0.07) °C and 60.09 (0.02) °C in the absence and presence of calcium, respectively) compared to oryzin in the presence of calcium (Fig. 9a). Calcium at less than 10 μM caused no variation of the transition temperature, but the T_m_ steadily increases with increasing calcium from 20 μM. A ΔTm = 1 °C is achieved at 312 uM calcium. Since oryzin is secreted it may be exposed to an as high as 2 mM extracellular calcium environment^43^ suggesting its physiological significance. While the difference in T_m_ is small (ΔT_m_ = 2 °C), these changes in T_m_ in the presence of calcium compares to what has been previously observed in subtilisin with similar magnitudes^44^. Similarly, calcium increases the transition temperature at which the protein aggregates resulting in an increase in the unfolding ΔTm of the protein. Oryzin begins to aggregate at 57.40 (0.12) °C and 59.39 (0.06) °C in the absence and presence of calcium, respectively, following the trend observed in the thermal unfolding experiment. As the effect of calcium is to structurally stabilize the molecule, the questions as to where the calcium binding site locates and what its binding means to the protein fold, are to be solved. To this end, we analyzed a third crystal form, oryzin-calcium, obtained with calcium as an additive in the crystallization cocktail.

The calcium ion is placed at the center of an almost regular octahedron formed by oxygen ligands (Fig. 9b). Three of these ligands belong to the protein: carbonyl groups of Pro298 and Ala300 and the carboxylate of Asp323. Four other oxygens are provided by water molecules indicating that the calcium site is fully exposed to the solvent. The apex of the octahedron is defined by the Pro298–Asp323 axis while the rest of binders lie on a plane equatorially. The oxygen-calcium distances are all about 2.3–2.5 Å, typical for calcium-oxygen contacts.

**Figure 10:**
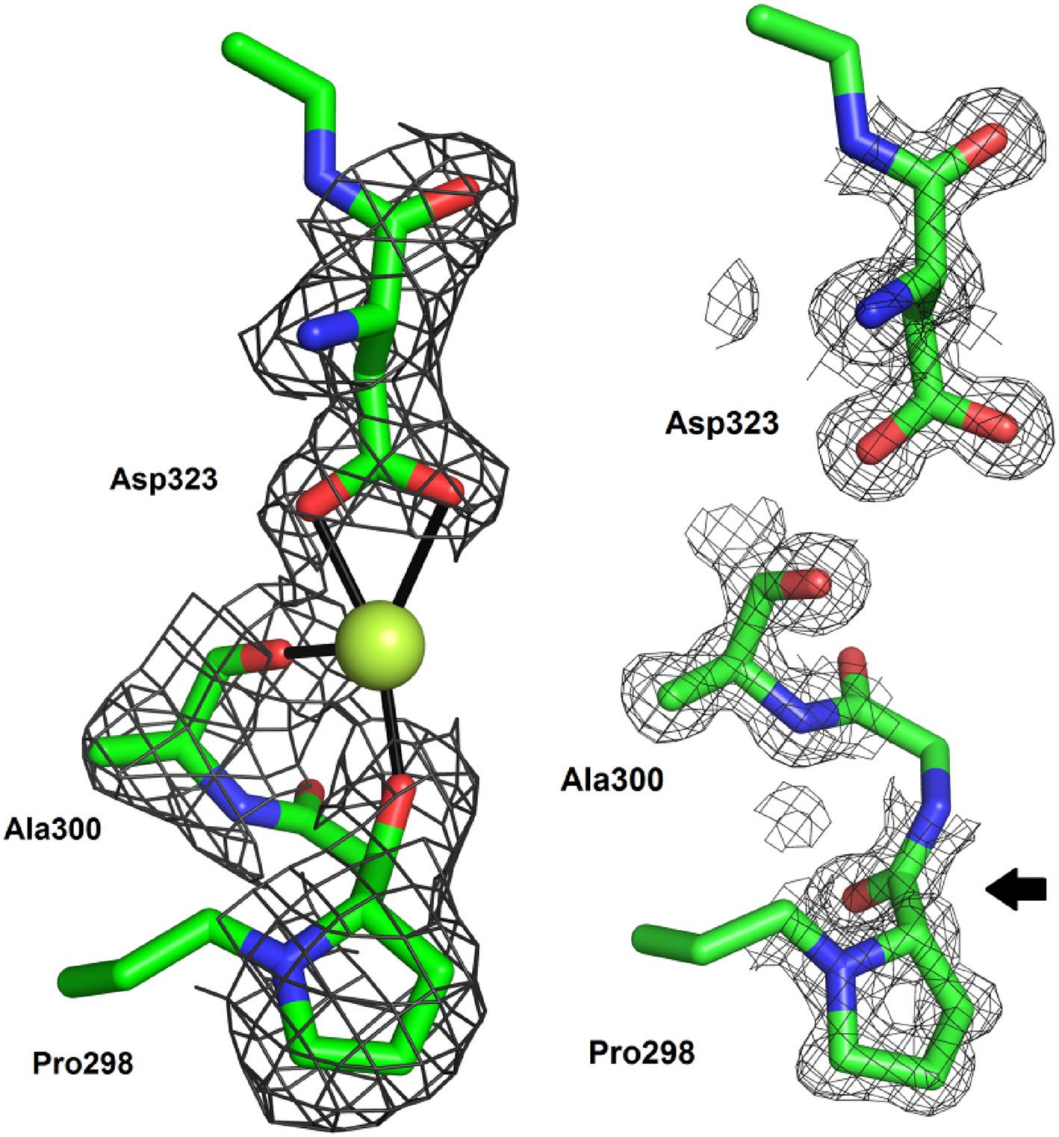
Backbone remodeling at Pro298-calcium binding site. Detail of binding interactions for calcium (water molecules omitted for clarity). Pro298 carbonyl oxygen provides one of oxygen ligands to the calcium, but its carbonyl is flipped (arrow) when unbound. Atoms rendered as sticks in green (carbon), blue (nitrogen), and red (oxygen). Calcium (sphere, lemon). Electron density map (grey mesh) contoured at the 1.0-σ level.

The oryzin-calcium crystal shows that a calcium site is in a turn between sheets 6 and 7 located opposite to the catalytic cleft face (Fig. 10). An overlay between oryzin and oryzin-calcium reveals that calcium binding has a minimal effect on overall fold (rmsd alignment of 0.35 Å along 282 Cαs). However, the largest offset is at position 298 (rmsd of 2.1 Å between Pro298 Cα). This calcium binding site is created by backbone rearrangements in oryzin at Pro298 and stabilized by hydrogen bonding interactions between Glu269 and the amide N atom of Pro298, and the amide N and side chain of Asn299. Interestingly, while Asp323 is conserved among subtilisins, Glu269 is not. For example, in *P. cyclopium* subtilisin, an alanine occupies the equivalent place of Glu269, the proline is further from Asp323, and its carbonyl O atom is replaced by a water molecule. The calcium ions are in the same position in both structures. Moreover, in human pro-protein convertase, the calcium site relates to the oryzin calcium site (they are further away to 2.5 Å). In the unbound form (Fig. 10, arrow), the backbone at Pro298–Asn299 bond is flipped outward of the turn and cannot form a binding site for calcium.

## Discussion

We report on the first crystal structure of *Aspergillus fumigatus* oryzin. This structure shows a product-trapped catalytic domain with its self-cleaved pro-domain peptide segment marking the enzyme's substrate prime binding pocket preferences. Analysis of the complex with the pro-peptide and modeling illustrates oryzin's S4′–S4 substrate binding pockets differ from the typical subtilisin-like α,β-hydrolases. In complex to an irreversible inhibitor, we label and confirm the identity of the catalytic machinery around the catalytic serine (Ser349). Combining crystallography and in vitro assays, our work confirms that oryzin binds calcium, that calcium stabilizes the enzyme, that the metal binding site is rather flexible and solvent exposed further from of the catalytic machinery. Thermal unfolding analysis combined with crystallographic data shows that the binding of calcium results in local structural changes, without detriment for the overall fold. Together with earlier observations our work suggests that enzyme activity changes are due to extra cleavage events that would result in changes in the protein integrity or because of products of hydrolysis directly modulating the catalytic mechanism by physical hindrance. Further work will be needed to address the complexities of this serine hydrolase substrate recognition properties and progression to its mature state. Diseases caused by *Aspergillus* spp. remain a challenging problem^45^ and oryzin is well suited for target-based drug development^46^. In the human genome, serine proteases are a predominant class of enzymes^47^ making the design of *Aspergillus*-specific inhibitors a difficult endeavor. Structure-based screening in exploratory research of new inhibitors is well established^48–50^ and may assist in the design effort. Combined with the present structural work it will help progress research into new agents to block progression of aspergillosis.

## Experimental procedures

### Oryzin cloning, expression, and purification

A plasmid for expression of the *A. fumigatus* sequence [UniProtKB^27^ ID: P28296; *Neosartorya fumigata* (strain ATCC MYA-4609/Af293/CBS 101355/FGSC A1100)] encoding a truncated protein lacking the 20 amino acids from the secretion signal and containing a C-terminal His_6_-tag cloned into a pD444 vector with ampicillin resistance and IPTG inducible T5 promoter was purchased as a synthetic gene (ATUM, Newark, CA, USA). After transformation of protease deficient *E. coli* strain BL21(DE3) [New England BioLabs, Ipswich, MA, USA], transformants were grown in lysogeny broth (Thermo Fisher Scientific, Waltham, MA, USA) containing ampicillin (100 µg/ml) at 37 °C in a shaking incubator at 225 rpm. The culture was grown until an optical density of 0.6–0.8 was reached and then transferred to a shaking incubator at 16 °C for 1 h before induction with 1 mM IPTG (G-Biosciences, St. Louis, MO, USA). After 3–4 h post induction, the culture was spun down at 10,000×*g* for 60 min at 4 °C, resuspended in 1/50 culture volume of buffer (150 mM NaCl, 16 mM Na_2_HPO_4_, 4mM NaH_2_PO_4_ [pH 7.3]), and, after addition of lysozyme and Triton X-100, incubated in a rotator shaker at 4 °C for 10 min. Cells were lysed by sonication in a Fisherbrand Model 705 Sonic Dismembrator (ThermoFisher Scientific) three or four times for 30 s each on ice, and the lysate was cleared by centrifugation at 12,000×*g* for 60 min at 4 °C. The supernatant was mixed with 1 ml of Ni–NTA resin (Qiagen, MD, USA), incubated in a rotator shaker at 4 °C for 30 min, and the resin was applied on a plastic column. After washing with 10 bed volumes of 20 mM Tris–HCl, 500 mM NaCl, 20 mM imidazole, pH 8.5, elution of the His_6_-tagged protein was performed with an imidazole gradient, 1 bed volume of 10–500 mM imidazole, stepwise by gravity. The purity of the eluted fusion protein was analyzed by SDS-PAGE (NuPAGE, Thermo Fisher Scientific). We attempted to capture the intact uncleaved zymogen by using acidic pH buffers, but we consistently obtained the separate polypeptides (judged by SDS-PAGE) suggesting that the cleavage products must have appeared very early during protein expression. Fractions containing pure protein as determined by SDS-PAGE analysis were loaded onto a Superdex 75 column for size-exclusion chromatography using a Akta Pure (Cytiva, Marlborough, MA, USA) system. The protein was concentrated to 30 mg/ml using Amicon Ultra 10 kDa MWCO spin filters (Millipore Sigma), in buffer 0.1 M MES, 0.05 M NaCl, pH 5.6, and aliquots stored at − 80 °C. For chemically denatured protein purification, oryzin lysate was incubated with 2.2 M GuHCl (as determined by chemical unfolding experiments) for 1 h at room temperature to allow for chemical unfolding of the protein. Protein was further purified following the protocol from above.

### Crystallization, data collection, structure solution and refinement

Crystals were obtained using sitting-drop vapor diffusion for screening and sitting- or hanging-drop vapor diffusion for optimization at 6 °C or 16 °C. Crystallization experiments were setup using a Douglas Oryx8 Nanodrop dispensing robot (Douglas Instruments Ltd, East Garston, United Kingdom). Diffraction-quality crystals were obtained from a solution containing 20% (w/v) polyethyleneglycol 550 monomethylether, 10% (w/v) polyethyleneglycol 20,000, sodium nitrate, sodium dihydrogen phosphate, ammonium sulfate (each 0.03 M), in 0.1 M MES/Imidazole buffer at pH 6.5. Crystals were harvested and cryocooled by plunging in liquid nitrogen with or without addition of cryoprotectant (polyethyleneglycol 200 or 1,2-ethanediol). Crystals were screened for their diffracting power at 100 K at beamlines BL14-1 and BL12-2^51^ at the Stanford Synchrotron Radiation Lightsource (SSRL) at SLAC National Accelerator Laboratory (Menlo Park, CA, USA). The best diffracting sample was up to a Bragg spacing of 1 Å and data was collected taking advantage of the macroscopic properties of the crystal. To mitigate radiation damage, we employed a helical data collection strategy on a rod-shaped crystal using fine slicing by translating the microfocused beam along the longest edge of the crystal. The crystal is in space group P2_1_2_1_2_1_ with cell dimensions a: 60.60 Å, b: 70.76 Å, c: 78.55 Å; α, β, γ: 90° with one polypeptide chain in the asymmetric unit. Solving the phase problem using high-quality diffraction data to find one single polypeptide chain consisting of 382 aminoacids at ~ 1 Å resolution proved to be practicable using a method to place idealized peptide fragments via molecular replacement iterated with automated cycles of density modification. Phasing of the 1.06 Å data was with fragment location with ideal polyalanine α-helices as search fragments. Calculations were performed with ARCIMBOLDO_LITE^52^, a method which has previously been shown to successfully phase a 385-residues target protein at a lower resolution. This method uses Phaser^53^ for searching combined with density modification and autotracing with SHELXE^54^. ARCIMBOLDO_LITE runs in two macrocycles: first, Phaser sequentially places all requested copies of the search model(s) and partial structures are ranked and optimized with SHELXE. Similar solutions are clustered; then, top-ranked substructure solutions (according to top LLG and CC; a CC > 25% indicates a possibly solved structure) are selected and subjected to density modification and autotracing with SHELXE. A promising substructure comprising 8 α-helices was expanded to trace a polyalanine-only model encompassing 367 residues (out of 382 from sequence). This solution gave a final CC of 49.89 and was subsequently chosen for refinement. This partial model was completed by successive rounds of manual adjustment and model building with COOT^55^ and refinement with REFMAC5^56^. The electron density map was of excellent quality and allowed for unambiguously tracing the pro-domain, residues Arg27–Asp121 (21–26 missing), while the catalytic domain could be traced fully from Ala122–Ala403. Refinement progressed to convergence and reached an excellent agreement to the experimental data (see Table S1 for crystal data quality parameters and refinement statistics). Additional molecules, namely formate, imidazole, and polyethyleneglycol were added in later stages of refinement. To test bond geometry hypotheses, we used SHELXL^57^ at various stages of refinement. Solvent water molecules were first assigned based on their hydrogen bonding properties. To prepare other samples for crystallization, either 10 mM PMSF or CaCl_2_ were added to the protein buffer before concentration. Oryzin-calcium was obtained from a crystallization condition containing 12.5% (w/v) PEG 1000, 12.5% (w/v) PEG 3350, 12.5% (v/v) MPD; L-glutamate, DL-alanine, glycine, DL-lysine, and DL-serine (each 0.02 M), in 0.1 M MOPS/HEPES, pH 7.5, with CaCl_2_ as an additive. The crystal is in space group P2_1_2_1_2_1_ with cell dimensions a: 59.25 Å, b: 66.07 Å, c: 86.79 Å; α, β, γ: 90° with one polypeptide chain in the asymmetric unit. Data at 1.65 Å Bragg spacing was collected at SSRL beamline BL12-2. The condition for the 1.06 Å crystal was repeated to obtain oryzin-PMS containing PMSF as an additive. The crystal is in space group P2_1_ with cell dimensions a: 51.09 Å, b: 75.21 Å, c: 87.92 Å; α, γ: 90°, β, 101.30° with two polypeptide chains in the asymmetric unit. Data at 1.55 Å Bragg spacing was collected at SSRL beamline BL12-2. Further crystals were grown using CaCl_2_ as an additive to collect anomalous data at 7 keV (1.7715 Å). The 1.06 Å crystal structure was used as the search model for molecular replacement of these derivatives with Phaser. See Tables S1–4 for crystal data quality parameters and refinement statistics. Data was reduced with XDS^58^, scaled with SCALA^59^, and analyzed with different software routines within the CCP4 suite^60^. Graphics were rendered with Pymol^30^.

### Molecular alignment, modeling, and docking

UniProtKB entries used for alignment: I3R794, Halolysin, *Haloferax mediterranei* (strain ATCC 33500/DSM 1411/JCM 8866/NBRC 14739/NCIMB 2177/R-4) (*Halobacterium mediterranei*); P29143, Halolysin, *Natrialba asiatica* (strain ATCC 700177/DSM 12278/JCM 9576/FERM P-10747/NBRC 102637/172P1); P04072, Thermitase, *Thermoactinomyces vulgaris*; P00782, Subtilisin BPN', *Bacillus amyloliquefaciens* (*Bacillus velezensis*); P00780, Subtilisin Carlsberg, *Bacillus licheniformis*; Q3S3L6, Putative 36kDa protease, *Lysinibacillus sphaericus* (*Bacillus sphaericus*); U5J9E4, Peptidase_S8 domain-containing protein, Bacillus phage vB_BanS-Tsamsa; J9E4K3, Peptidase_S8 domain-containing protein, *Wuchereria bancrofti*; A0QQ47, Subtilase family protein, *Mycolicibacterium smegmatis* (strain ATCC 700084/mc(2)155) (*Mycobacterium smegmatis*); P59996, Proprotein convertase subtilisin/kexin type 9, *Rattus norvegicus* (Rat); Q8NBP7, Proprotein convertase subtilisin/kexin type 9, *Homo sapiens* (Human); A0A2R2JFW8, Protease, *Penicillium cyclopium*; P06873, Proteinase K, *Parengyodontium album* (*Tritirachium album*); Q01471, Serine protease, *Purpureocillium lilacinum* (*Paecilomyceslilacinus*); B8N106, Alkaline protease 1, *Aspergillus flavus* (strain ATCC 200026/FGSC A1120/IAM 13836/NRRL 3357/JCM 12722/SRRC 167); P12547, Alkaline protease 1, *Aspergillus oryzae* (strain ATCC 42149/RIB 40) (Yellow koji mold); P28296, Alkaline protease 1, *Neosartoryafumigata* (strain ATCC MYA-4609/Af293/CBS 101355/FGSC A1100) (*Aspergillus fumigatus*); D5LGB3, Alkaline protease 1, *Aspergillus versicolor*; A1CIA7, Alkaline protease 1, *Aspergillus clavatus* (strain ATCC 1007/CBS 513.65/DSM 816/NCTC 3887/NRRL 1/QM 1276/107); F2E033, Predicted protein, *Hordeum vulgare* subsp. vulgare (Domesticated barley); P87184, Alkaline protease 2, *Neosartorya fumigata* (strain ATCC MYA-4609/Af293/CBS 101355/FGSC A1100) (*Aspergillus fumigatus*); Q8GB52, Extracellular subtilisin-like serine proteinase, *Vibrio* sp. PA-44; Q3HUQ2, Proteinase K, *Serratia* sp. GF96; P80146,Extracellular serine proteinase, *Thermus* sp. (strain Rt41A); P08594, Aqualysin-1, *Thermus aquaticus*;P16397, Bacillopeptidase F, *Bacillus subtilis* (strain 168). Alignment was done with Seaview^38^. Structure comparisons were done with DALI server^31^. All molecular modeling was performed using Coot and the model coordinates refined against reference parameters with REFMAC5 (version 5.8.0135) until deviation from ideal bond length, bond angle, planar restraints, chiral volume, reached convergence and Ramachandran outliers were minimized (1.7%, 5 out of 347, of the total residues were outliers). Extension of the pro-domain beyond residue 121 was made exploiting the location of water molecules that hydrogen bond the protease to guide the tracing of the hydroxyl group of residues Thr124 and Thr125 and the main chain carbonyl of Gln126. The docking model was based on a synthetic triple-helix peptide containing a region from human collagen type III^61^. Molecular protein docking was made with Frodock version 2.0^62^. Docking was validated by using the pro-domain for a docking prediction and comparison to the experimentally determined structure. As a test, the pro-domain's coordinates were randomly oriented and then submitted to docking. The comparison with the experimentally determined binary complex showed an excellent agreement.

### Differential scanning fluorimetry and light scattering

Thermal unfolding measurements were performed using a Prometheus Panta instrument (NanoTemper Inc, South San Francisco, CA, USA). The thermal unfolding was on a linear thermal ramp from 25 to 95 °C to the sample while reading out fluorescence and turbidity to analyze thermal stability and aggregation. To assess protein aggregation and precipitation backreflection was used for measuring light intensity loss due to scattering. Data recorded by the backreflection optics is termed turbidity. The unfolding profile plots the 350nm/330 nm fluorescence ratio as a function of temperature. The ratio is a measure for a spectral shift in the fluorescence emission profile of tryptophan residues. Non-aggregated oryzin exhibits a turbidity signal of under 100 mAU and then sharply goes up on melting. The overall degree of aggregation is calculated according to the Beer–Lambert law and expressed in attenuation units (AU), plotted as milli-AU. Turbidity data was measured in parallel to the thermal unfolding experiment on the same capillaries. The cumulant analysis was used for dynamic light scattering (DLS) data analysis and to assess sample homogeneity. It models the autocorrelation function (ACF) using an average diffusion coefficient to obtain a single averaged hydrodynamic radius rH. Heating rate was with a temperature slope of 0.5 °C/min. Experiments performed by triplicate. Oryzin stock concentration was at 0.4 mg/ml in 0.05 M Hepes buffer, pH 7.5 with 0.1M NaCl. CaCl_2_ was used in serial dilutions between 50 mM and 1.2 µM. Additionally, MgCl_2_ was tested at concentrations between 6.25 and 50 mM, but the T_m_ for this metal ion was indistinguishable from protein control without metal. Tabulated data are the average value and standard deviation (calculated from all DLS acquisitions).

### Chemical unfolding

Chemical unfolding measurements were performed using Prometheus Panta (NanoTemper Inc., South San Francisco, USA). Guanidine hydrochloride (GuHCl) was used as the chemical denaturant with 30 µL dilutions from 6 to 0.25 M prepared in Tris–HCl buffer. A 0.5 mg/mL stock of oryzin in 0.2 M Tris–HCl buffer, pH 8.0, was added to each sample and incubated for 1 h at room temperature. The unfolding profile plots the 350 nm/330 nm fluorescence ratio as a function of denaturant concentration. The ratio is a measure for a spectral shift in the fluorescence emission profile of tryptophan residues. The c50, denaturant concentration in which 50% of the protein is unfolded, is calculated.

### Substrate-enzyme activity assay

The substrate used to determine enzymatic activity of oryzin was Suc-Ala-Ala-Pro-Phe-pNA (Suc-AAPF-pNA; Bachem Americas Inc., Torrance, CA, USA). Suc-AAPF-pNA 10 mM stock solutions were prepared in 0.2 M Tris–HCl buffer, pH 8.0, aliquoted, and stored at − 20 °C. Protein stocks were all diluted to the lowest concentration stock (0.2 mg/mL) using Tris–HCl, pH 8.0. Samples were prepared in a cuvette as 1 mL total volume as follows: 100 μL protein of interest, 50 μL of Suc-AAPF-pNA substrate, 850 μL Tris–HCl, pH 8.0 buffer. A blank was prepared using 50 μL of Suc-AAPF-pNA substrate and 950 μL Tris–HCl, pH 8.0 buffer. Samples were measured at 410 nm using a Nanodrop 2000c spectrophotometer (ThermoFisher Scientific, Wilmington, DE, USA) at 0, 0.02, 0.08, 0.2, 0.5, 1, 2, 3, 4, 5, 6, 21, 23, 24 h, making sure to blank before each measurement. Substrate-enzyme activity could also be visualized by a color change from clear to yellow.

### Supplementary Information

Supplementary Information.

## Data Availability

The atomic coordinates and structure factors generated during the current study are available in the Protein Data Bank^63^ with accession codes: oryzin (PDB: 8GKO), oryzin-PMS (PDB: 8GKP), and oryzin-calcium (PDBs: 8GKQ and 8U45). All other data are contained in this manuscript.
