# Supplementary material for: Targeting Aspergillus allergen oryzin with a chemical probe at atomic precision
Source: Sci Rep. 2023 Oct 20;13:17926. doi: 10.1038/s41598-023-45028-z (PMC10589352; doi:10.1038/s41598-023-45028-z)
Supplement: Supplementary file 1 — Supplementary Information. [file 41598_2023_45028_MOESM1_ESM.docx]

**Supporting Information**

Targeting *Aspergillus* Allergen Oryzin with a Chemical Probe at Atomic Precision

Olivia Pattelli^1,2^, Dinh Dinh Ly Diec^1,2^, Wanting Guo^1,2^, Silvia Russi^3^, Daniel Fernandez^1,2*^

^1^SarafanChEM-H, Stanford University, Stanford, CA 94305, USA;

^2^Macromolecular Structure Knowledge Center, Stanford University, Stanford, CA 93405;

^3^Structural Molecular Biology Group, Stanford Synchrotron Radiation Lightsource (SSRL), SLAC National Accelerator Laboratory, Menlo Park, CA 94205

*Correspondence should be addressed to D.F.

Email: [danilo@stanford.edu](mailto:bertozzi@stanford.edu)

*Protein purification*


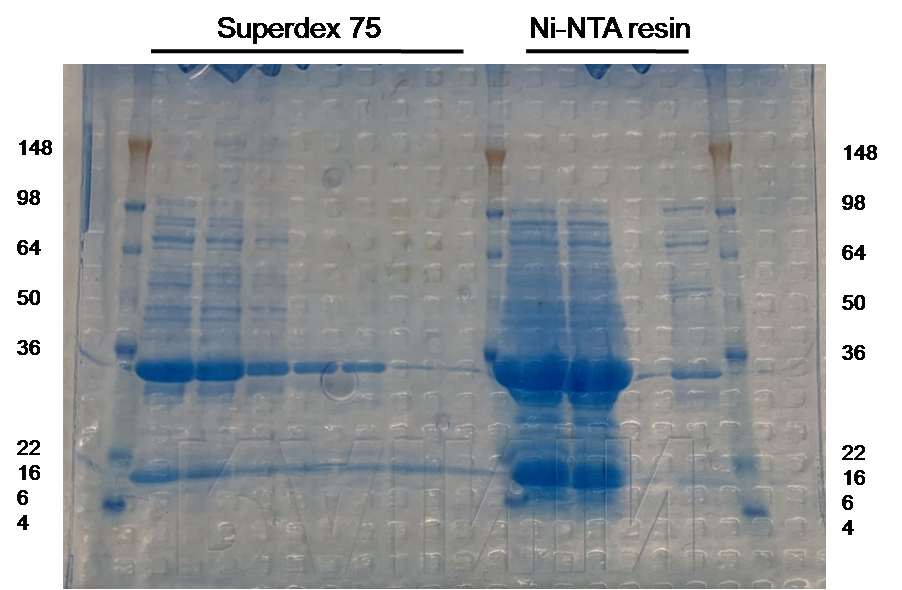


**Figure S1**. Oryzin purification. *Left*, size exclusion running glycine 0.1 M, pH 2.5. *Right*, affinity purification on Ni-NTA beads. Molecular weight ladder in KDa.


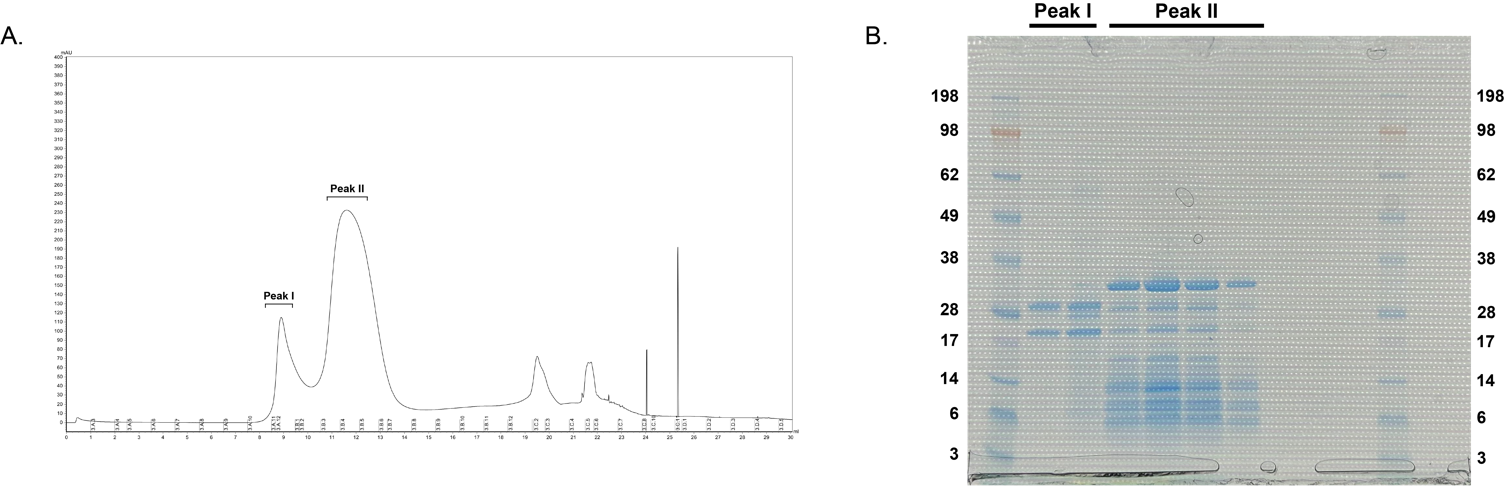


**Figure S2.** Oryzin purification of chemically denatured lysate. ***(a)*** Size exclusion chromatography trace of oryzin showing two distinct protein peaks. This was after incubation of lysate with 2.2 M GuHCl and purification using immobilized metal affinity chromatography. ***(b)*** SDS-Page gel of fractions contained in each peak of FPLC trace. Molecular weight ladder in KDa.

*Crystallographic Data*

**Table S1. Data collection and refinement statistics**

| **Data collection for oryzin** |  |
| --- | --- |
| Beamline  Wavelength (Å)  Space group | SSRL BL12-2  0.97946  P2_1_2_1_2_1_ |
| Cell dimensions |  |
| *a*, *b*, *c* (Å) | 60.60, 70.76, 78.55 |
| α, β,γ(°) | 90.00, 90.00, 90.0 |
| Solvent content (%)^a^  Resolution (Å)^b^  No. of reflections/unique | 38.0  39.28(1.06)  549,701 / 142,896 |
| *R*_merge_^c^ | 0.076(1.125) |
| *I*/σ*I* ratio and CC_1/2_^d^ | 8.6(0.7) / 0.998(0.420) |
| Completeness (%)^e^ | 93.5(65.2) |
| Redundancy^f^ | 3.8(2.0) |
| **Refinement** |  |
| Resolution (Å) | 30.6-1.06 |
| No. of reflections/test set | 135,789 / 7,013 |
| *R*_work_ / *R*_free_^g^ | 14.0 / 16.0 |
| F_obs_-F_calc_correlation^h^  No. of atoms | 0.98 |
| Protein | 5,810 |
| Ligand/ion | 10 (imidazole) / 40 (ethyleneglycol) / 12 (formate) / 1 (sodium) |
| Water | 483 |
| *B*-factors |  |
| Protein | 11.7 |
| Ligand/ion | 31.5 (imidazole) / 41.1 (ethyleneglycol) / 17.6 (formate) / 12.0 (sodium) |
| Water | 26.3 |
| R.m.s. deviations |  |
| Bond lengths (Å) | 0.009 |
| Bond angles (°) | 1.384 |
| Ramachandran statistics^i^ |  |
| Most favored regions (%) | 99.7 (323 out of 324 non-proline/non-glycine residues) |
| Disallowed regions (%) | 0.3 |

^a^Ratio of the volume of the asymmetric unit to the molecular weight of all protein molecules in the asymmetric unit

^b^Value in parentheses is for the outermost resolution shell 39.28-1.06 (1.12-1.06)

^c^Reliability factor for symmetry-related reflections calculated as: *R*_merge_ = Σ_hkl_Σj=1 to N | I_hkl_ – I_hkl_ (j) | / Σ_hkl_Σj=1 to N I_hkl_ (j), where N is the redundancy of the data. In parentheses, the cumulative value at the highest-resolution shell

^d^Ratio of mean intensity to the mean standard deviation of the intensity over the entire resolution range and correlation coefficient for random half-datasets for merged data

^e^Fraction of measured reflections to possible observations at the resolution range

^f^Number of measurements of individual, symmetry unique reflections

^g^Average deviation between the observed and calculated structure factors calculated as: *R*_work_ = Σ_hkl_ ||F_obs_| - |F_calc_|| / Σ_hkl_ |F_obs_|, where the F_obs_ and F_calc_ are the observed and calculated structure factor amplitudes of reflection hkl. *R*_free_ is equal to *R*_factor_ but for a randomly selected 5.0% subset of the total reflections that were held aside throughout refinement for cross-validation

^h^Correlation coefficient between observed and calculated structure factor amplitudes

^i^According to Procheck for non-proline and non-glycine residues

**Table S2. Data collection and refinement statistics**

| **Data collection for oryzin-PMS** |  |
| --- | --- |
| Beamline  Wavelength (Å)  Space group | SSRL BL12-2  0.97946  P2_1_ |
| Cell dimensions |  |
| *a*, *b*, *c* (Å) | 51.09, 75.22, 87.93 |
| α, β,γ(°) | 90.00, 101.3, 90.0 |
| Solvent content (%)^a^  Resolution (Å)^b^  No. of reflections/unique | 37.0  37.61(1.55)  355,823 / 91,847 |
| *R*_merge_^c^ | 0.064(0.945) |
| *I*/σ*I* ratio and CC_1/2_^d^ | 10.9(1.4) / 0.999(0.563) |
| Completeness (%)^e^ | 97.3(95.6) |
| Redundancy^f^ | 3.9(3.8) |
| **Refinement** |  |
| Resolution (Å) | 30.2-1.55 |
| No. of reflections/test set | 87,267 / 4,546 |
| *R*_work_ / *R*_free_^g^ | 15.3 / 20.6 |
| F_obs_-F_calc_correlation^h^  No. of atoms | 0.96 |
| Protein | 5,653 |
| Ligand/ion | 5 (sulfate) / 7 (PEG) / 20 (PMS) / 33 (formate) / 3 (sodium) |
| Water | 414 |
| *B*-factors |  |
| Protein | 24.6 |
| Ligand/ion | 57.1 (sulfate) / 55.8 (PEG) / 39.2 (PMS) / 42.4 (formate) / 23.8 (sodium) |
| Water | 32.3 |
| R.m.s. deviations |  |
| Bond lengths (Å) | 0.013 |
| Bond angles (°) | 1.515 |
| Ramachandran statistics^i^ |  |
| Most favored regions (%) | 99.7 (642 out of 644 non-proline/non-glycine residues) |
| Disallowed regions (%) | 0.3 |

^a^Ratio of the volume of the asymmetric unit to the molecular weight of all protein molecules in the asymmetric unit

^b^Value in parentheses is for the outermost resolution shell 37.61-1.55 (1.63-1.55)

^c^Reliability factor for symmetry-related reflections calculated as: *R*_merge_ = Σ_hkl_Σj=1 to N | I_hkl_ – I_hkl_ (j) | / Σ_hkl_Σj=1 to N I_hkl_ (j), where N is the redundancy of the data. In parentheses, the cumulative value at the highest-resolution shell

^d^Ratio of mean intensity to the mean standard deviation of the intensity over the entire resolution range and correlation coefficient for random half-datasets for merged data

^e^Fraction of measured reflections to possible observations at the resolution range

^f^Number of measurements of individual, symmetry unique reflections

^g^Average deviation between the observed and calculated structure factors calculated as: *R*_work_ = Σ_hkl_ ||F_obs_| - |F_calc_|| / Σ_hkl_ |F_obs_|, where the F_obs_ and F_calc_ are the observed and calculated structure factor amplitudes of reflection hkl. *R*_free_ is equal to *R*_factor_ but for a randomly selected 5.0% subset of the total reflections that were held aside throughout refinement for cross-validation

^h^Correlation coefficient between observed and calculated structure factor amplitudes

^i^According to Procheck for non-proline and non-glycine residues

**Table S3. Data collection and refinement statistics**

| **Data collection for oryzin-calcium** |  |
| --- | --- |
| Beamline  Wavelength (Å)  Space group | SSRL BL12-2  0.97946  P2_1_2_1_2_1_ |
| Cell dimensions |  |
| *a*, *b*, *c* (Å) | 59.25, 66.07, 86.79 |
| α, β,γ(°) | 90.00, 90.00, 90.0 |
| Solvent content (%)^a^  Resolution (Å)^b^  No. of reflections/unique | 39.0  36.27(1.65)  240,711 / 40,727 |
| *R*_merge_^c^ | 0.077(1.068) |
| *I*/σ*I* ratio and CC_1/2_^d^ | 6.8(1.0) / 0.997(0.510) |
| Completeness (%)^e^ | 98.1(99.7) |
| Redundancy^f^ | 3.0(3.0) |
| **Refinement** |  |
| Resolution (Å) | 30.6-1.65 |
| No. of reflections/test set | 38,658 / 2,028 |
| *R*_work_ / *R*_free_^g^ | 20.7 / 24.7 |
| F_obs_-F_calc_correlation^h^  No. of atoms | 0.96 |
| Protein | 2,799 |
| Ligand/ion | 1 (calcium) / 7 (PEG) / 8 (ethanediol) / 3 (formate) |
| Water | 162 |
| *B*-factors |  |
| Protein | 29.2 |
| Ligand/ion | 23.7 (calcium) / 42.4 (PEG) / 45.6 (ethanediol) / 41.5 (formate) |
| Water | 36.2 |
| R.m.s. deviations |  |
| Bond lengths (Å) | 0.012 |
| Bond angles (°) | 1.467 |
| Ramachandran statistics^i^ |  |
| Most favored regions (%) | 99.7 (320 out of 321 non-proline/non-glycine residues) |
| Disallowed regions (%) | 0.3 |

^a^Ratio of the volume of the asymmetric unit to the molecular weight of all protein molecules in the asymmetric unit

^b^Value in parentheses is for the outermost resolution shell 36.27-1.65 (1.74-1.65)

^c^Reliability factor for symmetry-related reflections calculated as: *R*_merge_ = Σ_hkl_Σj=1 to N | I_hkl_ – I_hkl_ (j) | / Σ_hkl_Σj=1 to N I_hkl_ (j), where N is the redundancy of the data. In parentheses, the cumulative value at the highest-resolution shell

^d^Ratio of mean intensity to the mean standard deviation of the intensity over the entire resolution range and correlation coefficient for random half-datasets for merged data

^e^Fraction of measured reflections to possible observations at the resolution range

^f^Number of measurements of individual, symmetry unique reflections

^g^Average deviation between the observed and calculated structure factors calculated as: *R*_work_ = Σ_hkl_ ||F_obs_| - |F_calc_|| / Σ_hkl_ |F_obs_|, where the F_obs_ and F_calc_ are the observed and calculated structure factor amplitudes of reflection hkl. *R*_free_ is equal to *R*_factor_ but for a randomly selected 5.0% subset of the total reflections that were held aside throughout refinement for cross-validation

^h^Correlation coefficient between observed and calculated structure factor amplitudes

^i^According to Procheck for non-proline and non-glycine residues

**Table S4. Data collection and refinement statistics**

| **Data collection for oryzin-calcium** |  |
| --- | --- |
| Beamline  Wavelength (Å)  Space group | SSRL BL14-1  1.77115  P2_1_ |
| Cell dimensions |  |
| *a*, *b*, *c* (Å) | 42.91, 94.59, 52.92 |
| α, β,γ(°) | 90.00, 103.2, 90.0 |
| Solvent content (%)^a^  Resolution (Å)^b^  No. of reflections/unique | 54.1  38.21(2.10)  105,471 / 22,839 |
| *R*_merge_^c^ | 0.111(0.874) |
| *I*/σ*I* ratio and CC_1/2_^d^ | 5.5(1.1) / 0.993(0.331) |
| Completeness (%)^e^ | 95.1(82.6) |
| Redundancy^f^ | 4.6(3.7) |
| **Refinement** |  |
| Resolution (Å) | 30.53-2.10 |
| No. of reflections/test set | 21,626 / 1,158 |
| *R*_work_ / *R*_free_^g^ | 19.1 / 22.9 |
| F_obs_-F_calc_correlation^h^  No. of atoms | 0.96 |
| Protein | 2,762 |
| Ligand/ion | 1 (calcium) / 42 (PEG) / 1 (chlorine) |
| Water | 118 |
| *B*-factors |  |
| Protein | 40.1 |
| Ligand/ion | 32.6 (calcium) / 56.3 (PEG) / 59.1 (chlorine) |
| Water | 43.9 |
| R.m.s. deviations |  |
| Bond lengths (Å) | 0.017 |
| Bond angles (°) | 1.767 |
| Ramachandran statistics^i^ |  |
| Most favored regions (%) | 99.7 (320 out of 321 non-proline/non-glycine residues) |
| Disallowed regions (%) | 0.3 |

^a^Ratio of the volume of the asymmetric unit to the molecular weight of all protein molecules in the asymmetric unit

^b^Value in parentheses is for the outermost resolution shell 38.21-2.10 (2.21-2.10)

^c^Reliability factor for symmetry-related reflections calculated as: *R*_merge_ = Σ_hkl_Σj=1 to N | I_hkl_ – I_hkl_ (j) | / Σ_hkl_Σj=1 to N I_hkl_ (j), where N is the redundancy of the data. In parentheses, the cumulative value at the highest-resolution shell

^d^Ratio of mean intensity to the mean standard deviation of the intensity over the entire resolution range and correlation coefficient for random half-datasets for merged data

^e^Fraction of measured reflections to possible observations at the resolution range

^f^Number of measurements of individual, symmetry unique reflections

^g^Average deviation between the observed and calculated structure factors calculated as: *R*_work_ = Σ_hkl_ ||F_obs_| - |F_calc_|| / Σ_hkl_ |F_obs_|, where the F_obs_ and F_calc_ are the observed and calculated structure factor amplitudes of reflection hkl. *R*_free_ is equal to *R*_factor_ but for a randomly selected 5.0% subset of the total reflections that were held aside throughout refinement for cross-validation

^h^Correlation coefficient between observed and calculated structure factor amplitudes

^i^According to Procheck for non-proline and non-glycine residues
